# Rising Trends in Wrestling-associated Injuries in Females Presenting to US Emergency Departments

**DOI:** 10.5811/westjem.2020.9.48490

**Published:** 2020-12-16

**Authors:** Connor Hoge, Kevin Pirruccio, Olivia G. Cohen, John D. Kelly

**Affiliations:** * University of Cincinnati College of Medicine, Department of Orthopaedics and Sports Medicine, Cincinnati, Ohio; † University of Pennsylvania, Perelman School of Medicine, Department of Orthopaedic Surgery, Philadelphia, Pennsylvania

## Abstract

**Introduction:**

Wrestling is one of the fastest-growing sports among females in the United States (US). However, female wrestling injuries remain poorly characterized. In this study we describe historical and projected national estimates of female wrestling injuries, and compare injury characteristics with those of male wrestlers.

**Methods:**

We queried the National Electronic Injury Surveillance System (NEISS) database (2005–2019) to compare national weighted estimates and injury characteristics of male vs female wrestlers presenting to US emergency departments (ED) and projected annual female wrestling injuries expected by 2030.

**Results:**

Our analyses demonstrated a significant (P < 0.001) increase in female wrestling injuries between 2005 (N = 1500; confidence interval [CI], 923 – 2,078) and 2019 (N = 3,404; CI 2,296 – 4,513). Linear regression (R2 = 0.69; P < 0.001) projected 4,558 (CI, 3104 – 6033) such injuries in 2030. Of female wrestling injuries 50.1% (CI, 44.1 – 56.2) occurred in patients 14–18 years of age. Compared with age-matched males, female wrestlers were significantly less likely to present with fractures (Female [F]: 10.6%; CI 7.5% – 13.7%; Male [M]: 15.7%; CI 14.7% – 16.7%; P = 0.003) or head/neck injuries (F: 18.5%; CI 13.2% – 23.9%; M: 24.6%; CI 23.2% – 26.0%; P = 0.018), and significantly more likely to present with strains/sprains (F: 48.8%; CI, 41.2% – 56.3%; M: 34.4%; CI 31.6% – 37.1%; P < 0.001).

**Conclusion:**

Males and females possess distinctly unique physiology and anatomy, such as variances in ligamentous and muscular strength, which may help to explain differences in wrestling injury characteristics. Prompt management of injuries and specific training strategies aimed at prevention may help to reduce the projected increase of female wrestling-associated injuries as the popularity of the sport continues to rise.

## INTRODUCTION

Wrestling has long been regarded as one of the most physically taxing sports in the world, with an injury rate of nearly 70 per 1,000 athletic exposures, which is second only to football.^1^ In the United States (US), wrestling has historically been a male-dominated sport, and injuries among male wrestlers have been well described.^2^ Among females, wrestling is one of the fastest-growing sports: participation has increased for 30 consecutive years with a parallel rise in female-only competitive tournaments.^3^ Uniquely, female and male wrestlers often practice and compete together at the high school level, yet national regulations vary between the genders. For example, requirements for body composition state that male and female wrestlers must maintain body fat percentages greater than 7% and 12%, respectively.^4^ Fat-free body mass has been associated with strength, power, and elite performance among both male and female wrestlers, and different body fat requirements may therefore contribute to variations in performance and injuries among wrestlers.^5^–^7^

Despite increased participation, female wrestling injuries remain poorly characterized. For instance, female wrestlers at the elite and Olympic levels have lower observed injury rates than males, with no significant differences in injury sites or severities. However, sample sizes in such studies are low and do not include youth wrestlers.^8^ Even broader epidemiological investigations on wrestling-associated injuries have historically excluded female wrestlers from their analyses altogether due to underpowered sample sizes.^9^ With the recent surge in popularity of wrestling among female participants, data from larger cohorts of female athletes is now available to better inform injury prevention strategies, and training and rehabilitation programs for all wrestlers.

In this study we describe historical and projected national estimates of female wrestling-associated injuries and compare injury characteristics among females and males during the study period. We hypothesized that an increase in wrestling-associated injuries among females would be observed over the study period, and that female wrestlers would sustain lower proportions of severe injuries such as fractures and concussions compared with males.

## METHODS

We retrospectively identified cases of wrestling-associated injuries in the National Electronic Injury Surveillance System (NEISS), which is maintained by the US Consumer Product Safety Commission (CPSC). The NEISS database functions to oversee and document product- or activity-related injuries presenting to US EDs; it is publicly available, deidentified, and published annually on a freely accessible governmental website. Importantly, it is a nationally representative probability sample of designated hospital EDs stratified by hospital size and geographic location, from which weighted national estimates and sampling errors for queried injuries may be derived. Various reliable, reproducible epidemiologic studies on injury-related ED visits have been published using this database.^10^,^11^ Specific information pertaining to collection methodologies and quality control precautions are available on the CPSC webpage.^12^

In this study we queried each yearly sample in the NEISS database between 2005–2019, both inclusive, for injuries classified as associated with the sport of wrestling (Product Code: 1,270 – “Wrestling (activity/apparel/equipment)”). A total of 16,404 unique cases were identified in the NEISS database during this period, which amounted to 590,803 weighted national estimates of wrestling-associated injuries presenting to US EDs. Of note, we excluded the years prior to 2005 due to an overall low case number of female wrestling-associated injuries, which made annual statistics unstable. Next, free-text case narratives were searched to identify and exclude cases unrelated to the sport of wrestling. These included the following activities: *sumo wrestling, mud wrestling, World Wrestling Entertainment, Inc. wrestling*, and any description of a patient wrestling with a sibling, friend, or parent in a non-sports setting (eg, *on the couch*, *horsing around at home*, etc.). We identified 472 cases unrelated to the sport of wrestling, leaving 15,932 unique cases amounting to 569,813 weighted national estimates of wrestling-associated injuries presenting to US EDs for our final analyses.

**Population Health Research Capsule**

What do we already know about this issue?

Wrestling is one of the fastest-growing sports among females in the United States (US). However, female wrestling injuries remain poorly characterized.

What was the research question?

What is the projected increase of female wrestling injuries and how do they compare to male injury characteristics?

What was the major finding of the study?

Female wrestling injuries are projected to increase, and they suffer different injury characteristics than males.

How does this improve population health?

An understanding of the projected trend and injury characteristics will allow implementation of appropriate injury prevention and emergency care to female wrestlers.

We calculated all weighted national estimates, standard errors (SE), and 95% confidence intervals (CI) by using the *svyset* function in Stata/IC 15.1 statistical software (StataCorp LLC, College Station, TX).^13^ Significance of trends in the total national survey estimates was determined using adjusted Wald tests, given the use of weighted survey data. *P* values < 0.05 (2-sided) were considered significant.

## RESULTS

**Figure 1:**
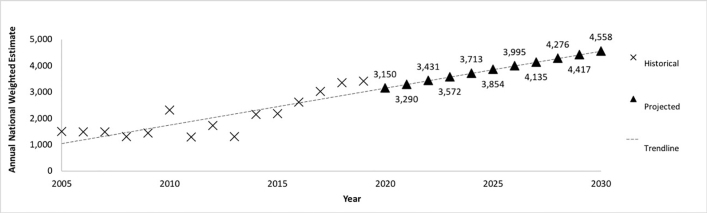
Historical and projected weighted national estimates of female patients presenting to US emergency departments with wrestling-associated injuries, 2005–2030.

Annual national estimates of wrestling-associated injuries among female participants between 2005 and 2019 are shown in Table 1. The national number of female patients per year presenting to US EDs increased significantly (*P* < 0.001) from 2005 (N = 1,500; CI, 923–2,078) to 2019 (N = 3,404; CI, 2,296–4,513). In Figure 1, linear regression (R^2^ = 0.69; *P* < 0.001) projected female wrestling injuries to reach 4,556 (CI, 3,104–6,033) by 2030.

The demographic characteristics of female patients presenting to US EDs with wrestling-associated injuries between 2005–2019 are shown in Table 2. More than half of females sustaining wrestling-associated injuries were 14–18 years of age (50.1%; CI, 44.1–56.2%). The majority of female patients identified as White (51.4%; CI, 43.3–59.6%). Race was not specified for 25.4% (CI, 16.4–34.5%) of patients, and 98.7% (CI, 97.9–99.5%) of patients were treated and released from the ED.

Injury characteristics, including body parts affected and final diagnoses for patients 14–18 years of age, are stratified by patient gender and reported in Table 3. Males sustained significantly (*P* = 0.018) greater proportions of head and neck injuries (24.6%; CI, 23.2–26.0%) compared with females (18.5%; CI, 13.2–23.9%). No other significant differences in affected body parts were demonstrated (*P* > 0.05). When comparing diagnoses, females sustained significantly (*P* < 0.001) greater proportions of sprains and strains (48.8%; CI, 41.2–56.3%) compared with males (34.4%; CI, 31.6–37.1%). In addition, males sustained significantly (*P* = 0.003) greater proportions of fractures (15.7%; CI, 14.7–16.7%) as compared with females (10.6%; CI, 7.5–13.7%).

Table 4 ranks the top five most common body parts affected in sprain or strain injuries among patients 14–18 years of age presenting to US EDs with wrestling-associated injuries during the study time period, stratified by patient gender. No significant differences (*P* > 0.05) were found in body parts sprained or strained between male and female wrestlers. The most commonly sprained or strained body part in both female (17.1%; CI, 12.8–21.5%) and male (20.3%; CI, 19.0–21.7%) wrestlers was the shoulder.

## DISCUSSION

Our findings demonstrate that female wrestling-associated injuries presenting to US EDs increased significantly over time. Recently, national estimates have more than doubled, rising from 1,500 in 2005 to 3,404 in 2019. Furthermore, the incidence of annual female wrestling-associated injuries is projected to be greater than 4,550 by 2030. To our knowledge, this is the first study on the prevalence and characteristics of female wrestling-associated injuries in the US. As the popularity of the sport continues to grow, an in-depth understanding of injury characteristics in female wrestlers will be integral to the development and implementation of risk minimization strategies in practice and competitions.

The most likely explanation for the significant and increasing trend of wrestling-associated injuries among female athletes in our study is rising participation, particularly at the high school level. According to the National Federation of State High School Associations (NFHS), 4,334 high school females in the US participated in wrestling during the 2004–2005 academic year.^14^ The most recent NFHS participation survey estimated 21,134 females participated in high school wrestling during the 2018–2019 academic year.^3^ This rise in participation is likely multifactorial, reflecting increased societal acceptance of female participation in a male-dominated sport and more opportunities for competition. Given that injury rates in wrestling are highest during competition as opposed to practice,^15^ the overall risk of wrestling-associated injuries in female participants may be rising as they are afforded more opportunities to compete at higher levels.^3^

Wrestling-associated injuries have been well characterized in male participants. For instance, the study by Myers and colleagues used the NEISS database to characterize wrestling-associated injuries in all participants aged 7–17 years old between 2000–2006.^9^ However, their analyses excluded female wresters because they only constituted 3.5% (5,998/173,604) of wrestling-related ED visits during the study period. Therefore, they were unable to report on any characteristics of wrestling-associated injuries in females. Nearly 15 years later, our analyses benefit from improved statistical power in order to compare and contrast wrestling-related injuries between male and female participants.

The vast majority (50.1%) of wrestling-related injuries in females occurred in high school-age athletes. Our comparisons reveal that female wrestlers in this age group were significantly less likely to sustain fractures than male wrestlers. Our supplemental analysis of all ages (Supplemental Table 1) found similar injury differences between males and females as the high school-age cohort, albeit a significantly higher rate of wrestling-related concussions by male athletes. Similar injury characteristics have been found in other sports, such as basketball and soccer: male participants generally suffer more fractures rather than strains/sprains compared with females.^16^ In wrestlers, this may be due to males using more high-risk takedown techniques, which inherently increase the risk of sustaining more severe injuries.^3^ In addition, female wrestlers were significantly more likely to sustain strains and sprains compared with male counterparts, which may be partly explained by differences in ligamentous strength and laxity. In general, females have more lax ligaments compared with males, allowing for greater flexibility.^17^ However, this makes the ligaments more prone to sustaining more strain and thus becoming injured. The incidence of female high school athletes suffering ligamentous knee injuries that require surgery is nearly double their male counterparts, illustrating this effect.^18^ Specifically in high school soccer, female athletes have been found to be up to 13.3 times more likely to suffer ligamentous knee injuries during competition that require surgery.^19^ Females have also been found to have decreased hamstring to quadriceps ratios, predisposing them to ligamentous knee injuries.^20^

The higher propensity for both male and female wrestlers to sustain strains and sprains relative to other injury types underscores the need for more targeted training measures that help ensure muscles remain both strong and flexible. For instance, training programs that include strength, balance, plyometric, and agility exercises have been found to significantly reduce ankle sprains and anterior cruciate ligament tears among female athletes.^21^,^22^ In addition, all injuries should be promptly reported to coaches, trainers, or team physicians so that proper care may be initiated expeditiously. Typically, it is recommended that first-degree strains be managed with rest, ice, compression, and elevation therapy, while second- and third-degree strains require evaluation by a physician. Inappropriate triaging or delays in management can aggravate injuries and predispose athletes to more severe diagnoses. Therefore, implementing these targeted interventions may help minimize the burden of strain- and sprain-related wrestling injuries while maximizing success on the mat.

Differences in injury characteristics between male and female wrestlers may also be explained by the number of athletic exposures. More skilled athletes typically stay on the mat longer in practice and competition, increasing their overall exposure to injury.^15^,^23^ Furthermore, a study by Kordi and colleagues found that the risk for fractures and dislocations was positively correlated with years of wresting experience and age of sport initiation.^24^ Thus, as more female athletes are exposed to wrestling at earlier ages, the overall injury characteristics may begin to more closely resemble those of males given the inevitable increase in practice, skill, and injury exposure that has been previously demonstrated in the literature.^2^,^15^,^23^

## LIMITATIONS

There are several limitations to this study that are associated with use of the NEISS database. First, the data only include injuries that presented to US EDs. Patients with less acute injuries may have first presented to urgent care or primary care offices. Therefore, our wrestling-associated injury estimates most likely underreport the true national burden of said injuries, instead emphasizing the most severe cases. Second, the database does not code for multiple injuries in a single ED encounter. In such situations, the NEISS survey only codes for the single most severe injury. Thus, multiple injuries suffered by a single participant would not be captured. Third, there may be differences in rules, regulations, and wrestling styles that vary on a state-by-state or national level that were not accounted for. At the scholastic level nationally, both males and females wrestle *folkstyle*, while collegiately males wrestle *folkstyle* and females switch to *freestyle*. We did not have the statistical power to analyze injuries in this specific demographic and thus did not make conclusions on any differences in injury characteristics due to wrestling styles.

Although the number of female wrestling-associated injuries in all participants and the 14–18 age group was large enough to undertake this epidemiologic study, statistical power was limited when evaluating more granular comparisons of injuries between males and females by body part, diagnosis, or age group. It is possible that true differences between the types of strains and sprains were not identified due to inadequate statistical power. Thus, our ability to make more specific training and injury prevention recommendations based on certain body parts or diagnosis was limited.

## CONCLUSION

We predict that the incidence of wrestling-associated injuries in female participants will increase significantly over the next decade as the popularity of the sport continues to rise. Wrestling is unique compared with many other sports at the scholastic level in that males and females practice and compete together. This is the first study that reports on the youth female wrestling injury profile, and demonstrates that females sustain more strains and sprains than males. Although wrestling carries an inherent risk of injury, prompt management of these injuries combined with specific training strategies aimed at preventing them may help to reduce the inevitable increase of wrestling-associated injuries among female and male athletes alike.

## Supplementary Information



## Figures and Tables

**Table 1:** Weighted national estimates of female patients presenting to United States emergency departments with wrestling-related Injuries, 2005–2019.

Year	National case estimates	Standard error	95% Confidence interval
2019	3,404	558	2,296 – 4,513
2018	3,350	524	2,309 – 4,392
2017	3,014	419	2,182 – 3,846
2016	2,601	548	1,514 – 3,689
2015	2,178	453	1,279 – 3,078
2014	2,141	376	1,394 – 2,888
2013	1,291	293	710 – 1,873
2012	1,724	306	1,115 – 2,332
2011	1,279	315	655 – 1,904
2010	2,301	450	1,408 – 3,195
2009	1,428	259	914 – 1,942
2008	1,301	262	781 – 1,822
2007	1,473	243	991 – 1,955
2006	1,474	302	874 – 2,075
2005	1,500	291	923 – 2,078

**Table 2:** Demographic characteristics of female patients presenting to US emergency departments with wrestling-related injuries, 2005–2019.

Demographic variable	Percentage	Standard error	95% Confidence interval
Age (in years)			
≤4^a^	0.6%		
5 to 10	8.9%	1.7%	5.6% – 12.2%
11 to 13	16.4%	2.0%	12.3% – 20.4%
14 to 18	50.1%	3.0%	44.1% – 56.2%
19 to 25	11.3%	1.4%	8.5% – 14.1%
≥26	12.8%	1.9%	9.0% – 16.6%
Race			
White	51.4%	4.1%	43.3% – 59.6%
Black	10.9%	1.9%	7.2% – 14.7%
Hispanic	7.0%	1.4%	4.2% – 9.8%
Other^a^	5.2%		
Race not specified	25.4%	4.6%	16.4% – 34.5%
Disposition			
Treated and released	98.7%	0.4%	97.9% – 99.5%
Treated and admitted^a^	0.4%		

a The estimate is considered to be potentially unstable due to the number of unweighted cases from the sample frame totaling <20, the weighted national estimate totaling <1200, or coefficient of variation >33%. Therefore, no standard errors or confidence intervals are provided; the unstable percentage estimate is provided for reference purposes only. Variable results with sample frame totals <20 cases or percentages <0.1% were omitted from this table, resulting in percentage totals not necessarily summing to 100%.

**Table 3:** Injury characteristics for patients 14–18 years of age presenting to US emergency departments with wrestling-related injuries between 2005 and 2019, stratified by the reported gender of the patient.

Injury variable	Female		Male		P Value
	
	%	95% CI	%	95% CI	
Body Part					
Head & neck (Incl. face)	18.5%	13.2% – 23.9%	24.6%	23.2% – 26.0%	0.018
Shoulder	16.7%	12.4% – 20.9%	14.9%	13.9% – 15.9%	0.430
Knee	10.4%	6.8% – 14.0%	10.3%	9.6% – 11.1%	0.975
Elbow	10.9%	6.5% – 15.4%	7.0%	6.0% – 7.9%	0.089
Upper trunk	9.8%	5.9% – 13.7%	8.1%	7.2% – 9.1%	0.396
Lower arm^a^	1.3%		2.6%		
Lower trunk^a^	4.2%		3.0%		
Hand and wrist (Incl. fingers)	12.0%	7.9% – 16.2%	12.2%	11.0% – 13.5%	0.931
Foot and ankle (Incl. toes)	11.5%	7.4% – 15.7%	9.0%	8.3% – 9.8%	0.218
All other body parts^a^	4.6%		8.4%		
Diagnosis					
Strain sprain	48.8%	41.2% – 56.3%	34.4%	31.6% – 37.1%	<0.001
Fracture	10.6%	7.5% – 13.7%	15.7%	14.7% – 16.7%	0.003
Pain	13.2%	8.5% – 17.8%	9.7%	5.6% – 13.8%	0.154
Contusions/abrasions	11.5%	7.3% – 15.7%	12.3%	11.3% – 13.3%	0.712
Concussion or CHI	8.8%	4.9% – 12.7%	11.5%	10.5% – 12.5%	0.150
Dislocation^a^	4.6%		6.2%		
Laceration^a^	0.3%		5.6%		
All Other Diagnoses^a^	2.8%		5.0%		

a The estimate is considered to be potentially unstable due to the number of unweighted cases from the sample frame totaling <20, the weighted national estimate totaling <1200, or coefficient of variation >33%. Therefore, no standard errors or confidence intervals are provided; the unstable percentage estimate is provided for reference purposes only. Variable results with sample frame totals <20 cases or percentages <0.1% were omitted from this table, resulting in percentage totals not necessarily summing to 100%.

*CI*, confidence interval; *Incl*, including; *CHI*, closed head injuries including traumatic brain injuries.

**Table 4:** Top five most commonly sprained body parts in patients 14–18 years of age presenting to United States emergency departments with wrestling-related injuries between 2005–2019, stratified by the reported gender of the patient.

Body part sprained or strained	Female		Male		P Value
	
	%	95% CI	%	95% CI	
Shoulder	17.1%	12.8% – 21.5%	20.3%	19.0% – 21.7%	0.164
Foot and ankle (Incl. toes)	17.0%	11.0% – 23.0%	16.2%	14.6% – 17.7%	0.785
Hand and wrist (Incl. fingers)	16.6%	12.0% – 21.2%	15.1%	13.4% – 16.8%	0.564
Knee	15.5%	10.4% – 20.6%	16.1%	14.8% – 17.5%	0.803
Head and neck (Incl. face)	11.8%	6.9% – 16.6%	12.3%	10.6% – 13.9%	0.838

*CI*, confidence interval; *Incl*, including.
