# Supplementary material for: Rising Trends in Wrestling-associated Injuries in Females Presenting to US Emergency Departments
Source: West J Emerg Med. 2020 Dec 16;22(2):410–6. doi: 10.5811/westjem.2020.9.48490 (PMC7972392; doi:10.5811/westjem.2020.9.48490)
Supplement: Supplementary file 1 [file wjem-22-410-s001.docx]

**Supplemental Table 1.** Injury characteristics for patients of all ages presenting to US emergency departments with wrestling-related injuries between 2005 and 2019, stratified by the reported gender of the patient.

|  | Female | | | |  | Male | | | |  |
| --- | --- | --- | --- | --- | --- | --- | --- | --- | --- | --- |
| Injury Variable | % | 95% CI | | |  | % | 95% CI | | | P Value |
| Body part |  |  |  |  |  |  |  |  |  |  |
| Head & Neck (Incl. Face) | 18.8% | 14.6% | - | 23.0% |  | 23.2% | 22.0% | - | 24.4% | **0.033** |
| Shoulder | 14.4% | 11.0% | - | 17.8% |  | 14.7% | 13.9% | - | 15.6% | 0.921 |
| Knee | 11.4% | 8.7% | - | 14.1% |  | 9.4% | 8.8% | - | 10.1% | 0.159 |
| Elbow | 7.8% | 5.0% | - | 10.6% |  | 7.5% | 6.6% | - | 8.4% | 0.854 |
| Upper Trunk | 9.3% | 6.5% | - | 12.1% |  | 7.8% | 7.2% | - | 8.5% | 0.294 |
| Lower Arm* | 2.3% |  |  |  |  | 3.2% | 2.8% | - | 3.6% |  |
| Lower Trunk* | 5.7% |  |  |  |  | 3.3% | 2.9% | - | 3.8% |  |
| Hand & Wrist (Incl. Fingers) | 15.2% | 11.5% | - | 19.0% |  | 13.5% | 12.3% | - | 14.8% | 0.360 |
| Foot & Ankle (Incl. Toes) | 10.6% | 7.7% | - | 13.5% |  | 9.0% | 8.2% | - | 9.8% | 0.276 |
| All Other Body parts* | 4.5% |  |  |  |  | 8.3% |  |  |  |  |
| Diagnosis |  |  |  |  |  |  |  |  |  |  |
| Strain, Sprain | 45.4% | 38.1% | - | 52.7% |  | 33.8% | 30.8% | - | 36.7% | **0.001** |
| Fracture | 13.4% | 10.3% | - | 16.5% |  | 17.6% | 16.6% | - | 18.6% | **0.016** |
| Pain | 12.7% | 9.0% | - | 16.3% |  | 10.9% | 6.8% | - | 14.9% | 0.398 |
| Contusions/Abrasions | 12.1% | 9.1% | - | 15.2% |  | 12.1% | 11.1% | - | 13.2% | 0.998 |
| Concussion or CHI | 7.9% | 5.1% | - | 10.7% |  | 10.6% | 9.5% | - | 11.6% | **0.041** |
| Dislocation | 4.3% | 2.7% | - | 5.9% |  | 5.6% | 4.9% | - | 6.4% | 0.158 |
| Laceration* | 1.3% |  |  |  |  | 5.0% | 4.3% | - | 5.7% |  |
| All Other Diagnoses* | 3.0% |  |  |  |  | 4.4% |  |  |  |  |
| *The estimate is considered to be potentially unstable due to the number of unweighted cases from the sample frame totaling <20, the weighted national estimate totaling <1200, or coefficient of variation >33%. Therefore, no standard errors or confidence intervals are provided; the unstable percentage estimate is provided for reference purposes only. Variable results with sample frame totals <20 cases or percentages <0.1% were omitted from this table, resulting in percentage totals not necessarily summing to 100%.  *CI*, confidence interval; *Incl*, including; *CHI*, closed head injuries including traumatic brain injuries. | | | | | | | | | | |
